# Pattern-Anchored Diagnosis of Oral Epithelial Dysplasia: A Position Paper From COST Action INTERCEPTOR Working Group 2

**DOI:** 10.1007/s12105-026-01963-7

**Published:** 2026-09-03

**Authors:** Syed Ali Khurram, Keith D. Hunter, Edward W. Odell, Merva Soluk Tekkeşin, Mark Lingen, Pablo Agustín Vargas, Omar Kujan, Daniela Elena Costea, Paolo Bossi, Pierre Saintigny, Senada Koljenović

**Affiliations:** 1 Faculty of Health, School of Clinical Dentistry, University of Sheffield, Sheffield, UK; 2 Liverpool Head and Neck Centre, Molecular and Clinical Cancer Medicine, University of Liverpool, Liverpool, UK; 3 Head and Neck/Oral Pathology, Guy’s and St Thomas’ NHS Foundation Trust and King’s College London, London, UK; 4 Department of Oral Pathology, Faculty of Dentistry, Ankara University, Ankara, Turkey; 5 Department of Pathology, University of Chicago, Chicago, IL USA; 6 Oral Diagnosis Department, Piracicaba Dental School, State University of Campinas (UNICAMP), Piracicaba, Brazil; 7 Oral and Maxillofacial Biology & Diseases Research Stream, UWA Dental School, The University of Western Australia, Perth, WA Australia; 8 Gade Laboratory for Pathology and Norwegian Centre of Excellence for Cancer Biomarkers (CCBIO), Department of Clinical Medicine, Faculty of Medicine, University of Bergen, Bergen, Norway; 9 Department of Pathology, Haukeland University Hospital, Bergen, Norway; 10 Department of Biomedical Sciences, Humanitas University, Pieve Emanuele, Milan, Italy; 11 IRCCS Humanitas Research Hospital, Rozzano, Milan, Italy; 12 Department of Medical Oncology, Centre Léon Bérard, Lyon, France; 13 INSERM 1052, CNRS 5286, Centre Léon Bérard, CRCL, Univ Lyon, Université Claude Bernard Lyon 1, Lyon, France; 14 Department of Pathology, Antwerp University Hospital, Antwerp, Belgium; 15 University of Antwerp, Antwerp, Belgium

**Keywords:** Oral epithelial dysplasia, Oral potentially malignant disorders, Histopathological grading, Pattern recognition, Risk stratification, Proliferative verrucous leukoplakia

## Abstract

**Purpose:**

Oral epithelial dysplasia (OED) is the principal histopathological marker for malignant risk stratification in oral potentially malignant disorders (OPMDs). Current World Health Organisation (WHO) three-tier and other binary grading systems are limited by modest inter-observer reproducibility and suboptimal prognostic discrimination, particularly for architecture-predominant lesions where cytological atypia is inconspicuous. The most clinically consequential failure of current practice is not mis-grading but non-recognition of dysplasia altogether, with direct implications for patient surveillance and management.

**Methods:**

This position paper, arising from Working Group 2 of COST Action CA21140 INTERCEPTOR, synthesises evidence from inter-observer reproducibility studies, feature-based prognostic models, under-recognition studies, outcome data and computational pathology analyses to propose and contextualise a pattern-anchored, grade-retaining diagnostic framework for OED.

**Results:**

Five recognisable histological patterns are proposed: conventional/basaloid, keratinising/differentiated, verrucous, papillomatous, and HPV-associated, functioning as diagnostic anchors that capture the full morphological spectrum of OED, including architecture-predominant phenotypes that are disproportionately under-recognised in routine practice. Feature-based prognostic models demonstrate that architectural traits characterising these patterns (bulbous rete ridges, AUROC 0.74; cohesion loss, AUROC 0.73) predict malignant transformation more effectively than grade alone. Independent computational pathology analyses identify corresponding immune microenvironment and architectural signals as the strongest prognostic variables, providing convergent support.

**Conclusion:**

A two-step approach i.e. first identifying the dominant morphological pattern and then assigning grade within that pattern using existing WHO or binary criteria, offers a pragmatic, grade-retaining refinement compatible with current diagnostic frameworks and clinical pathways. The proposal is hypothesis-driven and requires prospective multicentre validation before wider adoption.

## Introduction

Oral epithelial dysplasia (OED) is the most widely accepted histopathological indicator of malignant risk in oral potentially malignant disorders (OPMDs) and remains central to patient surveillance, treatment decisions, and risk stratification [1]. The WHO three-tier grading system (mild, moderate, severe) and other binary grading systems, such as low-grade versus high-grade classification, continue to form the basis of diagnostic reporting worldwide [2, 3] and perform reasonably well at identifying lesions at the highest end of the risk spectrum.

However, although these systems have good negative predictive value, their positive predictive value is suboptimal, particularly in subtypes of OPMD that lack classical cytological features. The limitations of current grading systems are well recognised and reported in the literature. Inter- and intra-observer reproducibility remains modest even among experienced observers, with κ values of 0.22-0.31 for WHO three-tier grading and only marginal improvement to κ = 0.31 for binary grading [3, 4]. Prognostic discrimination within the mild-to-moderate range, in particular, is poor, with several studies demonstrating that moderate dysplasia does not behave as a biologically uniform intermediate category [5, 6]. Many OSCC arise from lesions not previously graded as severe dysplasia, including the much larger pool of lesions graded as mild or moderate [7], or from OPMD lesions not recognised as dysplastic at the time of biopsy [8].

A key diagnostic pitfall in routine practice is failure to recognise dysplasia in lesions where architectural disturbance predominates over cytological atypia. Keratinising, verrucous, and superficially maturing lesions may be under-interpreted as reactive or hyperkeratotic, particularly in the presence of inflammation [9, 10]. This under-recognition problem has substantial clinical consequences. Wils et al. demonstrated that explicit recognition of architecture-predominant dysplasia increased the hazard ratio for malignant transformation from 3.26 to 7.43 (95% CI: 3.2-17.1; *p* < 0.001) in a retrospective cohort [10], suggesting that a significant proportion of low-grade but high-risk lesions can be overlooked. Conversely, reactive epithelial atypia may be over-interpreted as dysplasia, contributing to false-positive diagnoses [11].

The WHO 5th edition expanded the list of dysplasia-associated histological features from 16 to 28 [12], including several architectural descriptors. However, without explicit weighting or synthesis guidance, this expansion may paradoxically increase interpretive variability. A recent global survey of 132 pathologists confirmed this and revealed poor recognition of key architectural features, with “irregular epithelial stratification” and “drop-shaped rete ridges” among the least-recognised criteria [13].

These challenges suggest that further refinement of grading thresholds alone is unlikely to resolve the core diagnostic problem. Instead, there is a need to improve recognition, contextualisation, and communication of dysplasia, particularly for non-specialist pathologists who evaluate the majority of oral biopsies in routine practice.

### Positioning Within COST Action INTERCEPTOR (WG2)

This position paper arises from Working Group 2 (WG2) of COST Action CA21140 INTERCEPTOR (INnovative Tools for Early Detection and Interception of Oral Cancer), an international network bringing together clinicians, pathologists, and scientists to improve early diagnosis and patient care in OPMDs. WG2 focuses specifically on disease characterisation and harmonisation of diagnostic practice. The present work reflects collective discussion within WG2 and addresses a shared unmet need identified across participating centres, namely the limitations of current grading systems for OED in routine diagnostic practice and the absence of a pragmatic, internationally applicable framework that improves dysplasia recognition whilst remaining aligned with WHO grading principles.

### Scope and Relationship to Existing Classification Systems

This paper addresses oral cavity mucosa and OPMDs. The oropharynx represents a biologically and aetiologically distinct site, particularly with respect to HPV-driven disease [14], and is not the focus of this paper.

The proposal is explicitly designed to operate within existing WHO and binary grading systems, not to replace them. Morphological patterns function as recognition anchors and contextual descriptors, assisting pathologists in identifying dysplasia across its full morphological spectrum before assigning a grade. Grading thresholds and terminology remain unchanged. The intent is to improve consistency of dysplasia recognition and interpretation whilst preserving compatibility with established WHO reporting and clinical pathways. The proposed patterns should be regarded as practical morphologic archetypes rather than discrete nosological entities as these are heuristic recognition anchors, not mutually exclusive biological categories.

Most of the individual patterns described here are independently recognisable in the existing literature: differentiated dysplasia, verrucous and PVL-associated morphology, and basaloid dysplasia have each been described before. What this position paper adds is not a new pattern taxonomy but the integration of these previously separate descriptions into a single two-step diagnostic workflow, pattern recognition followed by grading within pattern, together with an explicit position that existing WHO and binary grading systems are retained rather than replaced.

## Background and Evidence Synthesis

### Reproducibility and Real-World Diagnostic Practice

Inter-observer variability in OED grading has been demonstrated across multiple studies [11, 13, 15, 16].Meta-analyses show κ = 0.22-0.31 for WHO three-tier grading and κ = 0.31 for binary low/high-grade systems [3, 4], representing fair-to-moderate agreement by standard interpretation benchmarks [17]. It should be noted that systematic reviews in diagnostic pathology may systematically underrepresent studies with stronger methodological designs in favour of larger heterogeneous datasets; a well-recognised methodological constraint that may lead pooled analyses to underestimate the reproducibility achievable in structured or calibrated diagnostic settings. Nonetheless, even after calibration exercises, it is important to note that improved inter-observer agreement does not equate to improved prognostic performance: reproducibility is a statistical property of grading and whilst predictive value reflects clinical utility, these are independent variables, and modest gains in the former do not automatically translate to gains in the latter. Nonetheless, κ values rarely exceed 0.50, [15] and agreement deteriorates when pathologists return to independent practice [11].

Globally, the majority of oral cavity mucosal biopsies are reported by non-specialist histopathologists, often working in isolation without access to specialist oral and maxillofacial or head and neck pathology consultation [13]. In this context, calibration exercises, whilst valuable in research settings, are difficult to implement at scale because they require continuous reinforcement to maintain effect [11]. While formal morphological standards have been proposed in the past (most notably by Smith and Pindborg) [18], these descriptive frameworks have historically struggled to achieve widespread clinical adoption. Consequently, there is a clear need for more scalable, image-anchored definitions and recognisable morphological frameworks. By using structured reporting prompts and visual anchors, diagnostic systems can reduce interpretive drift without the need for the continuous, formal calibration that has limited previous standardisation efforts.

### Prognostic Limitations of Histological Grade in Isolation

Although higher grades of dysplasia are associated with increased malignant transformation risk, the predictive performance of grade alone remains modest. Systematic reviews demonstrate substantial overlap in transformation rates i.e. mild dysplasia transforms at 3.5-12% over 5 years, moderate at 4-18%, and severe at 11-36% [19]. Data from large population-based cohorts corroborate this modest prognostic discrimination: critically, in a cohort of nearly 5,000 leukoplakia patients, approximately 40% of oral cancers arose from lesions in which no dysplasia had been identified on prior biopsy [20],The wide confidence intervals reflect biological heterogeneity that conventional grading does not capture.

Binary grading (low versus high) shows only marginal improvement in prognostic discrimination compared with three-tier grading, with c-indices typically 0.52-0.58 in validation cohorts [3]. This distinction is important, as reproducibility is a statistical measure of observer agreement and is independent of clinical predictive performance. Consequently, interest has shifted toward identifying specific histological features with independent prognostic value.

Feature-based prognostic models demonstrate that individual architectural and cytological traits predict malignant transformation more effectively than grade alone. Features such as bulbous rete processes (AUROC 0.74), loss of epithelial stratification (AUROC 0.72), and loss of cohesion (AUROC 0.73) have been reported to predict malignant transformation in a six-point model [21]. A simplified two-point model combining bulbous rete ridges and cohesion loss achieved an AUROC of 0.774, predicting transformation better than conventional grading [21–23]. These findings are corroborated by independent multicentre studies demonstrating that individual feature counts, including loss of cohesion, prominent nucleoli, and intense inflammation, outperform global grade for predicting transformation [24, 25]. Importantly, these high-performing features tend to cluster within recognisable high grade morphological patterns rather than being evenly distributed across WHO grades.

### Architecture-Predominant Dysplasia and the Under-Recognition Problem

Studies show that explicit recognition of subtle, architecture-predominant or keratinising dysplasia substantially improves identification of lesions at risk of progression. In Wils et al.’s retrospective cohort (n = 84), inclusion of “differentiated dysplasia” (characterised by premature keratinisation, loss of cohesion in superficial layers, and basal atypia) increased the hazard ratio for malignant transformation from 3.26 (conventional dysplasia) to 7.43 (differentiated dysplasia; 95% CI: 3.2-17.1; *p* < 0.001) [10]. This ‘pattern’ has historically been under-diagnosed or considered reactive, leading to inappropriate reassurance and delayed intervention.

Subsequent studies have reinforced these findings. Wils et al. identified differentiated dysplasia in approximately 19% of an oral leukoplakia cohort, demonstrating substantially higher transformation rates compared with conventional dysplasia across risk-stratified subgroups [10, 26]. Kim et al. independently confirmed the characteristic histological features of this pattern in a Korean cohort, identifying it retrospectively in prior biopsies from patients who had subsequently progressed to invasive carcinoma, confirming its clinical relevance and the frequency with which it is overlooked in routine evaluation [27].

Support for pattern-based recognition comes from analogous developments in vulvar pathology. The recognition of differentiated vulvar intraepithelial neoplasia (d-VIN) transformed clinical practice by establishing that an architecture-predominant, p53-driven phenotype carries a 33-86% risk of progression to invasive carcinoma despite minimal cytological atypia [28, 29]. However, the oral situation differs in an important respect. In vulvar pathology the dramatic benefit came from separating HPV-driven from non-HPV-driven disease, with d-VIN representing a relatively rare minority phenotype. In the oral cavity, HPV-associated dysplasia is extremely uncommon, meaning that virtually all oral dysplasia involves some degree of keratinising or differentiated maturation at the basal or suprabasal level. The keratinising pattern in the oral cavity therefore does not represent a rare minority entity analogous to d-VIN, but rather a prevalent under-recognised phenotype, present to varying degrees in the majority of oral dysplastic lesions that warrants comparable vigilance and explicit recognition.

This overlap raises a direct question of how a dominant pattern is selected when a lesion displays mixed features, since a framework proposed to improve reproducibility must not simply relocate the reproducibility problem from grade to pattern. In practice, dominance is assessed by the proportion of the lesion occupied by the defining architectural features of a given pattern (for example, the extent of verrucous exophytic architecture relative to background keratinising change) rather than by the presence or absence of any single feature, since most lesions will show at least trace involvement by more than one pattern. Where two patterns are both substantially represented, Sect. "Grading Within Pattern" sets out that both should be reported and management guided by the higher-risk pattern; this dual-reporting option is intended to reduce, though not eliminate, the pressure to force a single dominant label onto genuinely mixed morphology. This dominance-assignment step is itself untested, and its inter-observer reproducibility is identified as a specific research priority in Sect. "Future Work" and acknowledged as unresolved in Sect. "Limitations".

Proliferative verrucous leukoplakia (PVL) exemplifies the limitations of conventional histological grading. It frequently demonstrates verrucous or keratinising architectural patterns with minimal cytological atypia, leading to initial diagnoses of hyperkeratosis or mild dysplasia [30–32]. Yet malignant transformation rates for PVL range from 44 to 46% in systematic reviews [30–32], with reported rates exceeding 60% in series with longer follow-up [33], regardless of the assigned grade. In these lesions, architectural pattern appears to be more important than cytological atypia in risk assessment. The term “verrucous hyperplasia” should not be used. Population-based data from a national cohort of over 3 million Taiwanese adults demonstrate that lesions diagnosed as oral verrucous hyperplasia carried the highest malignant transformation rate of all OPMDs (24.55 per 1,000 person-years, exceeding even erythroplakia), and specific genetic associations with transformation have been identified, confirming substantial biological risk [34, 35]. No validated histological diagnostic criteria for this entity have been established, and its continued use risks conveying a false sense of benignity. Note also that “exophytic oral verrucous hyperplasia” as a betel-associated lesion in Southeast Asian populations is a distinct entity and should not be conflated with PVL-associated verrucous lesions.

Histological features most strongly associated with PVL include verrucous architecture, church-spire keratosis, keratinising squamous proliferation, and basal compartment expansion [30–33]. Recognition of these patterns independent of grade warrants consideration of closer surveillance and should prompt discussion in a multidisciplinary context. Clonal mucosal change extending beyond the clinically apparent lesion (recognisable as areas of subtle architectural abnormality at histological margins), is under-recognised but represents a further indicator of field-level disease that pattern-based assessment may help to identify.

### Stromal Context and the Immune Microenvironment

Diagnostic criteria for OED focus on epithelial changes, but the stromal compartment provides important contextual information that can both accompany and complicate dysplasia recognition. Subepithelial lymphocytic bands and interface mucositis are frequently observed alongside OED and represent a well-recognised diagnostic pitfall. Cases reported as non-dysplastic have been shown to carry a prominent lichenoid infiltrate alongside a differentiated or keratinising epithelial pattern, leading to diagnoses of hyperkeratosis with lichenoid change rather than dysplasia [9, 36]. Pathologists should therefore recognise that although an interface inflammatory response is most often non-dysplastic, it does not exclude underlying epithelial dysplasia and should prompt careful assessment of epithelial architecture and maturation in the appropriate clinical context.

Chronic inflammation represents an additional and under-appreciated diagnostic challenge in OED assessment. It can simultaneously mask subtle epithelial abnormalities and induce reactive cytological atypia, diverting attention from the underlying disturbances in epithelial maturation and architectural organisation that characterise pattern-based dysplasia. In particular, lichenoid inflammatory changes characterised by a dense, band-like lymphocytic infiltrate at the epithelial-connective tissue interface can significantly complicate interpretation, as basal cell degeneration and regenerative epithelial responses may mimic or mask true dysplastic alterations [37]. This dual effect increases the risk of both under- and over-diagnosis, emphasising the importance of histopathological assessment in conjunction with clinical correlation. In this context, pattern recognition with its emphasis on architectural configuration rather than individual cytological features, may be particularly valuable in separating reactive from dysplastic epithelial change.

This stromal immune response has been reported to carry independent prognostic significance beyond its diagnostic relevance. Computational pathology analyses have demonstrated that peri-epithelial lymphocyte (PEL) density (concordance index 0.73) and intra-epithelial lymphocyte (IEL) scores (concordance index 0.67) can independently predict malignant transformation in OED, representing the quantitative read-out of the lichenoid stromal signal [38, 39]. These immune microenvironment features are particularly prominent in keratinising and verrucous pattern dysplasia, consistent with the biological distinctiveness of those phenotypes and supporting their importance in pattern-anchored assessment.

Non-inflammatory stromal remodelling, including fibroblast activation [40] and extracellular matrix changes [41], has been reported in OED. However, systematic review evidence does not support myofibroblast immunodetection as a standalone prognostic tool for classical OED [42]. Beyond diagnostic and prognostic relevance, stromal remodelling may exert direct biological effects on dysplastic cell survival. Activated fibroblasts and altered matrix may contribute to a permissive microenvironment for clonal expansion and transformation, adding further biological rationale for incorporating stromal context within a pattern-based framework. Stromal features are most appropriately incorporated as contextual signals rather than as independent classifiers, but the growing evidence from digital and computational pathology provides a practical avenue for their objective quantification.

## Rationale for a Pattern-Anchored Approach

The case for a pattern-anchored approach rests on five interconnected arguments. First, pattern assignment provides an immediate recognition anchor that reduces the risk of missing dysplasia when lesions do not conform to the conventional expectation of cytological atypia, directly addressing the under-recognition problem documented across multiple cohorts [9, 10, 22, 26]. Second, once a dominant pattern is identified, the pathologist can direct attention to the subset of features that tend to cluster within that pattern rather than evaluating all 28 WHO criteria simultaneously, reducing cognitive burden and improving consistency. Third, high-risk histological features do cluster within patterns: conventional/basaloid (bulbous rete, hyperchromasia, cohesion loss), keratinising (premature keratinisation, dyskeratosis, cohesion loss), verrucous (exophytic architecture, blunt rete ridges) [21, 22, 27] and this clustering is not evenly distributed across WHO grades. Fourth, pattern recognition is inherently teachable and lends itself to image-anchored exemplar resources particularly valuable for non-specialist pathologists; [13, 16] pattern-based frameworks may also reduce interobserver variability by directing observer attention to reproducible architectural anchors. Fifth, explicit pattern labelling in diagnostic reports conveys biological context alongside grade, which is especially important for architecture-predominant phenotypes where cytological grade alone may substantially understate risk. HPV-associated dysplasia is included for framework completeness, ensuring this biologically distinct entity is not miscategorised as conventional dysplasia despite its rarity in the oral cavity.

This proposed approach does not replace grading. Instead, it reframes grading within a clearer morphological context: dysplasia is first identified and characterised by pattern and then graded for severity within that pattern. This sequence of pattern recognition before grade assignment mirrors how pathologists work [43] and makes implicit diagnostic reasoning explicit and teachable.

## Proposed Pattern-Anchored Grading Framework

We propose a two-step descriptive approach:

- Identification of the dominant morphological pattern based on reproducible architectural and cytological features.
- Assignment of grade within that pattern using existing WHO or binary criteria, guided by knowledge of the association between specific features and pattern.

Example proposed diagnostic statements:

- Conventional (basaloid) pattern dysplasia, high-grade (severe dysplasia)
- Keratinising/differentiated pattern dysplasia, low-grade (mild dysplasia)
- Verrucous pattern dysplasia, low-grade [note: grade reflects atypia severity within pattern only; verrucous pattern risk communication is addressed in Sect. "Verrucous pattern".]

This preserves compatibility with existing systems whilst improving clarity, biological context, and communication of risk to treating clinicians.

### Recognised Histological Patterns

The following patterns have been reported in OED literature and are supported by published evidence and collective expert experience (Table 1 summarises defining architectural and cytological features, ancillary markers, clinical context, and risk for each pattern) [10, 11, 21–23, 26, 27, 29, 32].

**Table 1:** Synthesis of WHO 5th edition morphological criteria into distinct OED diagnostic patterns. This framework facilitates risk stratification by grouping isolated architectural and cytological features into reproducible biological signatures

Pattern	Architectural features	Cytological features	Clues/IHC	Clinical context	Risk
Conventional (basaloid)	Expansion of basal/proliferative compartment with basaloid cells more than a few layers thick; drop-shaped/bulbous rete; thin keratin layer, often separated or absent; loss of epithelial stratification; increased mitoses including suprabasal	High N:C ratio; anisonucleosis and hyperchromatism with irregularly shaped nuclei; prominent nucleoli; reduced keratinocyte cohesion with increased intercellular space in areas of pleomorphism	Ki67 basal→mid; ± p53 aberrancy (not a standalone risk modifier; may be early/non-transforming)	Erythroleukoplakia; ventrolateral tongue; floor of mouth; soft palate	*Tendency*: High; increases with compartment expansion and suprabasal mitotic activity *Pitfall*: Features may be absent in superficial or limited incisional biopsies *Action*: Excision or close surveillance guided by architectural and mitotic features
Keratinising (differentiated)	Surface ortho/parakeratosis; premature keratinisation (suprabasal eosinophilic cytoplasm); dyskeratosis (single cells/clusters lying deeply); broad/elongate rete processes; stratification relatively preserved; altered basal polarity	Subtle basal atypia; reduced keratinocyte cohesion in suprabasal and low prickle cell levels; cellular pleomorphism often subtle; pale nuclei can be seen, maturation often retained in upper layers	↓CK13, ↑CK17 (supportive) Highlights subtle atypia Basal Ki67	Homogeneous leukoplakia; lichenoid immune response at interface; buccal mucosa, lateral tongue, gingiva	*Tendency*: Real but frequently under-recognised. Broader risk-stratified cohort evidence at refs [10, 11] *Pitfall*: Prominent keratinisation masks basal atypia; commonly dismissed as reactive or hyperkeratotic *Action*: Heightened surveillance; MDT discussion where grade is elevated or atypia is present basally
Verrucous	Verruciform architecture; spectrum from slight keratotic peaking to church-spire projections; broad pushing rete processes aligned with keratin structure; blunt basement membrane	Minimal cytological atypia; mitoses restricted to basal/parabasal layers	Dense interface immune response	Associated with PVL spectrum (not diagnostic alone; correlate with clinical presentation) [44]. Gingiva, hard palate, alveolar ridge; topical tobacco users	*Tendency*: High within PVL clinical spectrum (> 60% at >7 years [33]); substantially lower for solitary lesions without multifocal disease *Pitfall*: Cytological grade alone understates biological risk. “Verrucous hyperplasia” should be avoided where atypia is present. Grade must not be reported in isolation *Action*: MDT discussion regardless of cytological grade for PVL; report pattern and grade together explicitly
Papillomatous	Exophytic papillary fronds; delicate fibrovascular cores; complex branching; endophytic growth component	Variable atypia; dyskeratosis; basal atypia at base of papillae. Distinguish from squamous papilloma (orderly maturation, no atypia, no endophytic growth)	–	Verrucous/non-homogeneous leukoplakia; any oral mucosal site. Superficial biopsies frequently under-sample	*Tendency*: Elevated, especially within the PVL spectrum; precise rates in solitary lesions not established *Pitfall*: Superficial biopsies under-sample; surface morphology may resemble squamous papilloma *Action*: Excisional biopsy where feasible to exclude occult carcinoma or higher-grade disease
HPV-associated	Acanthotic epithelium, often thick; marked expansion of basaloid/proliferative compartment; reduced surface maturation; karyorrhectic cells and apoptotic debris throughout basaloid layers	Diffuse atypia throughout; frequent atypical mitoses at all levels; karyorrhectic nuclei; no typical koilocytic change (viral replication usually focal if present)	HPV ISH/PCR (definitive; mandatory) p16 block+ (screening indicator only; not diagnostic alone in oral cavity)	Uncommon in oral cavity proper; ventrolateral tongue, FOM and buccal mucosa; younger patients, sometimes immunosuppressed	*Tendency*: Uncertain; morphologically equivalent to moderate/severe dysplasia. Formal grading criteria not yet defined. Included for framework completeness, to prevent this biologically distinct entity being miscategorised as conventional dysplasia *Pitfall*: p16 IHC alone is insufficient for diagnosis in the oral cavity; koilocytic change is not a reliable feature *Action*: Confirm HPV by ISH/PCR before assigning aetiology; manage as oral cavity cancer protocol

Grade severity within the assigned pattern using existing WHO or binary criteria. Report all co-existing patterns; manage to the highest-risk component. Mixed/two-tone lesions are common; classify and manage according to the highest-risk component. For verrucous pattern lesions, the diagnostic report must communicate both pattern and grade together, making explicit that the cytological grade alone does not reflect overall biological risk

Several references cited in this table (particularly refs [30, 33]) use the term ‘atypical verrucous hyperplasia’ (AVH) as a diagnostic category for exophytic verruciform lesions without overt conventional dysplasia. The Expert Consensus Guideline (ref 43) moved away from this terminology; population-based studies (refs [34, 35]) confirm these lesions carry substantial malignant risk and specific molecular changes. The present paper follows this evidence in recommending that ‘verrucous hyperplasia’ terminology be avoided

Conventional/basaloid [9, 19, 21–23, 45]. Keratinising (differentiated) [9, 10, 23, 26, 27, 46, 47].Verrucous and PVL [30–33, 43, 44].Papillomatous [32, 43]. HPV-associated [48, 49]. Immune microenvironment [38, 39, 50, 51]

OED, oral epithelial dysplasia; N:C, nuclear-to-cytoplasmic ratio; Ki67, marker of nuclear proliferation; CK13/CK17, cytokeratin 13/17; PVL, proliferative verrucous leukoplakia; FOM, floor of mouth; ISH, in situ hybridisation; IHC, immunohistochemistry

#### Conventional (Basaloid) Pattern

##### Defining Features

**Fig. 1:**
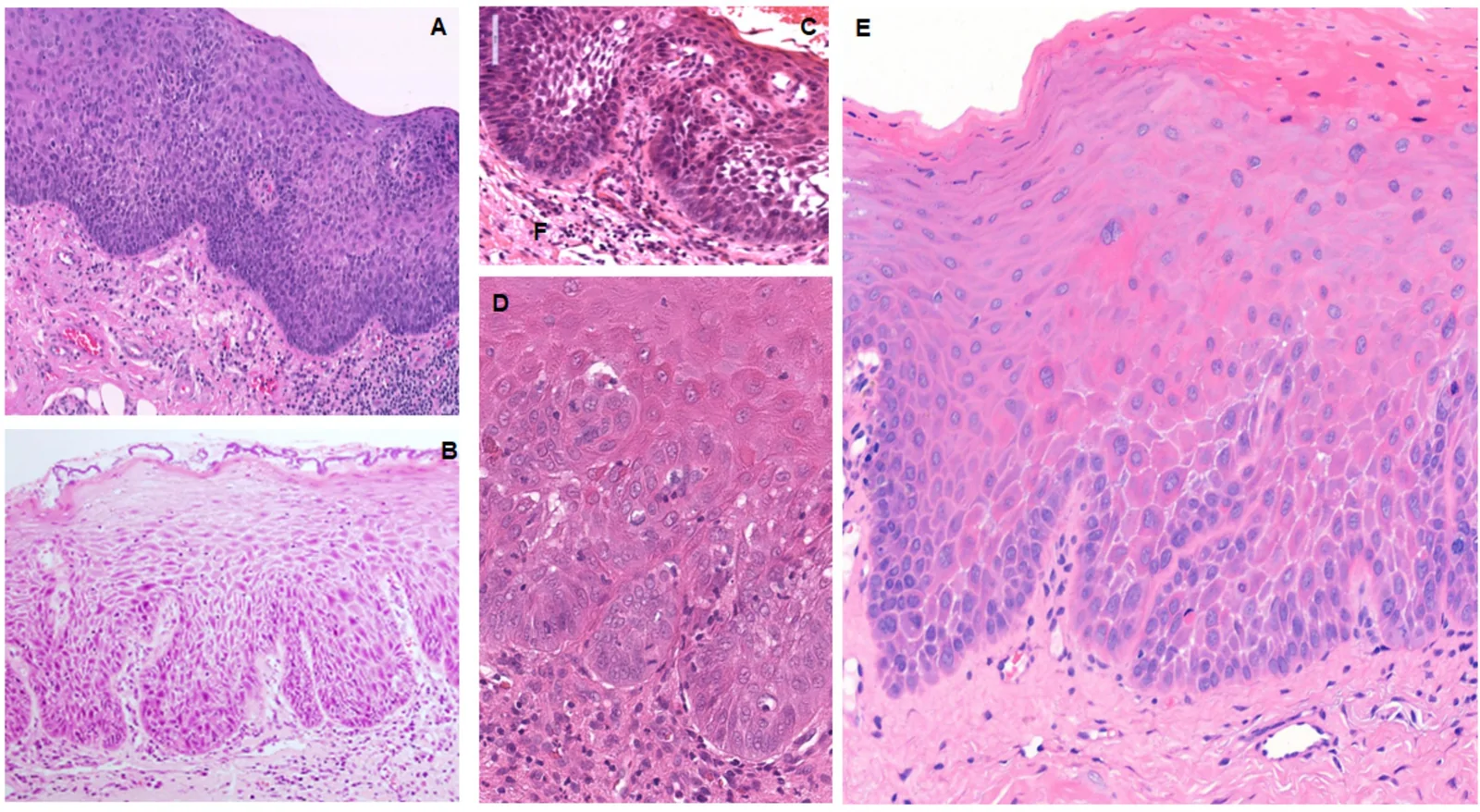
Conventional (basaloid) pattern. **A**-Expansion of the basal/proliferative compartment with nuclear crowding, hyperchromasia, and loss of maturation. **B**-Bulbous/drop-shaped rete ridges and **A**, **C**-a thin or absent surface keratin layer are characteristic; **D**-Basal cell nesting, **C**, **E**-cohesion loss with increased intercellular space in areas of pleomorphism may also be evident

The defining histological features of the conventional pattern include significant expansion of the basal and parabasal compartments, with basaloid cells extending more than a few cell layers above the basal layer, accompanied by nuclear crowding and the presence of more frequent and abnormal basal and suprabasal mitoses [19, 21]. Architectural alterations are characterised by the development of bulbous rete ridges, a discernible loss of cellular cohesion with increased intercellular space in areas of cytological pleomorphism, and a generalised disruption of epithelial maturation (Fig. 1).

##### Clinical Context

This morphology corresponds to the conventional form of dysplasia implicit in thirds-based grading systems and aligns with classical pathological descriptions of OED. This phenotype is typically associated with traditional risk factors, such as tobacco and alcohol consumption, and is a frequent histological finding in lesions presenting as erythroplakia.

##### Prognostic Features

The presence of bulbous rete ridges and loss of cellular cohesion have been reported as significant independent predictors of progression, with AUROC values of 0.74 and 0.73, respectively [21, 22]. Aberrant p53 immunoexpression may be observed in this pattern but should be interpreted with caution. p53 aberrancy can be an early and non-transforming event in OED, is not a driver in most conventional dysplasia, and should not be used in isolation to modify grade or upstage risk assessment.

#### Keratinising (Differentiated) Pattern

##### Defining Features

**Fig. 2:**
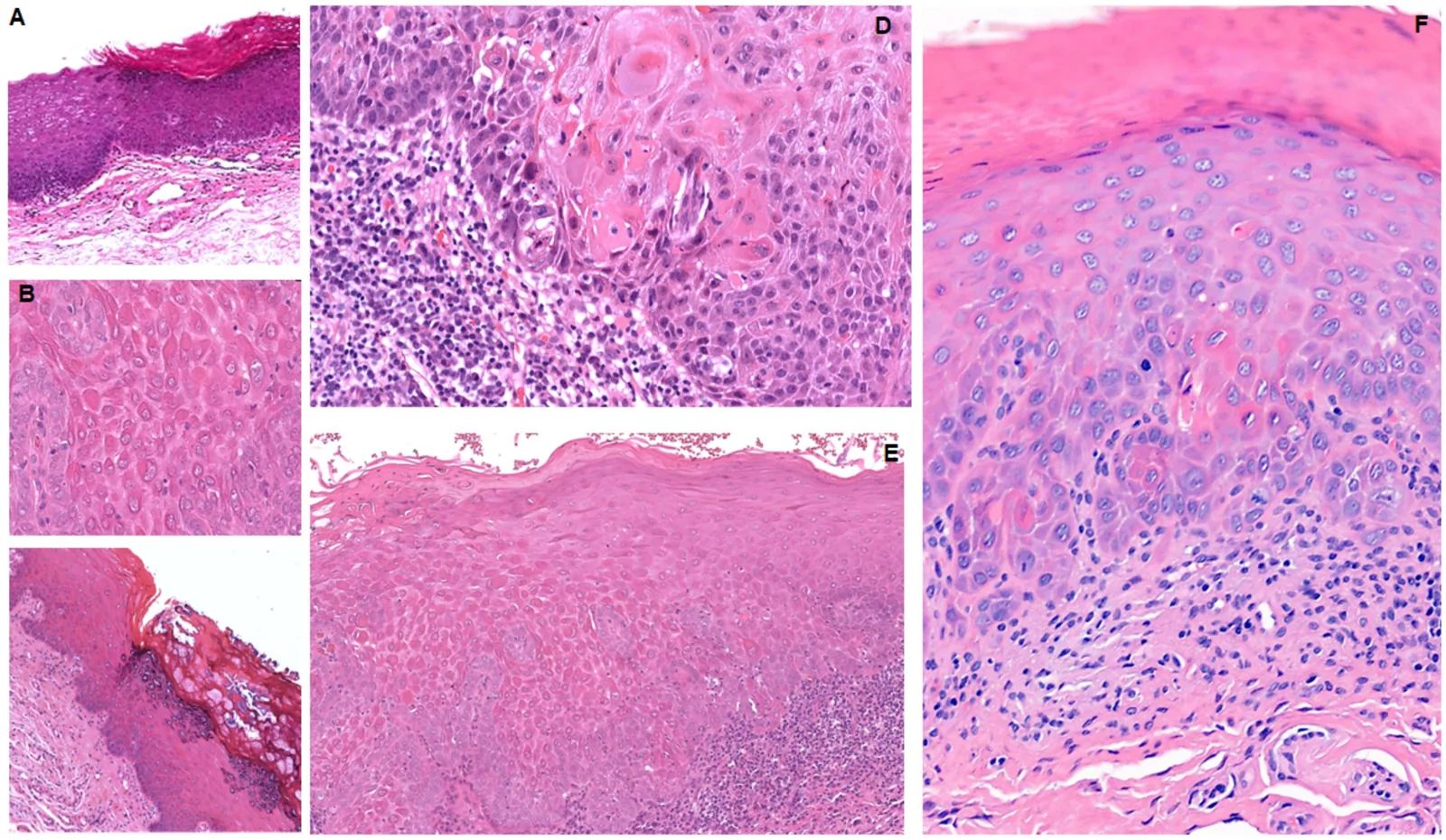
Keratinising (differentiated) pattern: **A**–**F**-Thick surface orthokeratosis; dyskeratosis and premature keratinisation in the spinous layer; **A**, **C**-preserved stratification with subtle basal atypia confined to the lower third in some instances; **D**–**E**-lichenoid interface immune response. **A**–**C**: The dominant keratinisation (and at times lack of hyperchromatic nuclei) can mask the subtle basal changes, making this pattern prone to under-diagnosis as hyperkeratosis

The keratinising pattern is characterised by prominent surface orthokeratosis or parakeratosis and two related but distinct forms of abnormal keratinisation within the spinous layer (Fig. 2). Premature keratinisation refers to the acquisition of eosinophilic cytoplasm from partial keratinisation in suprabasal cells that would not normally keratinise at that level. Dyskeratosis, by contrast, denotes more severely disrupted keratinisation producing single cells or small clusters lying deeply within the epithelium, separated from the normal maturation sequence, and often associated with loss of cohesion in the low prickle cell levels. In contrast to conventional dysplasia, epithelial stratification might be preserved in some cases. Cytological atypia is frequently subtle with often enlarged, pale nuclei/prominent nucleoli frequently accompanied by an underlying lichenoid-type lymphocytic infiltrate [10, 11, 23, 26].

##### Terminology and Conceptual Distinction

While some authors propose ‘Differentiated Dysplasia’ as a unique p53-driven entity, [10, 26] we contend that the broader designation of Keratinising Pattern offers superior clinical utility. It ensures that the pathologist is cognisant of the morphological paradox; by prioritising this pattern-based recognition, high-risk lesions are more reliably captured regardless of whether they fulfil the restrictive molecular criteria required for a formal diagnosis of ‘differentiated’ dysplasia.

##### Clinical Context and Diagnostic Pitfalls

Lesions exhibiting this pattern are frequently under-interpreted as reactive or inflammatory processes, yet they carry a substantial risk of malignant transformation, with a reported hazard ratio of 7.43 [10]. The primary diagnostic pitfall lies in a morphological paradox. The presence of dramatic keratinisation can mask subtle basal atypia, leading the observer to dismiss the lesion as benign. Pathologists must therefore maintain a high index of suspicion, actively seeking subtle basal alterations and dyskeratosis rather than passively excluding dysplasia on the basis of superficial maturation. Supporting immunohistochemical markers include loss of CK13 and gain of CK17 expression, consistent with the abnormal keratinocyte differentiation programme; these are helpful for highlighting subtle dysplastic changes (cytologic atypia) but are not specific for the differentiated dysplasia pattern.

#### Verrucous Pattern

##### Defining Features

**Fig. 3:**
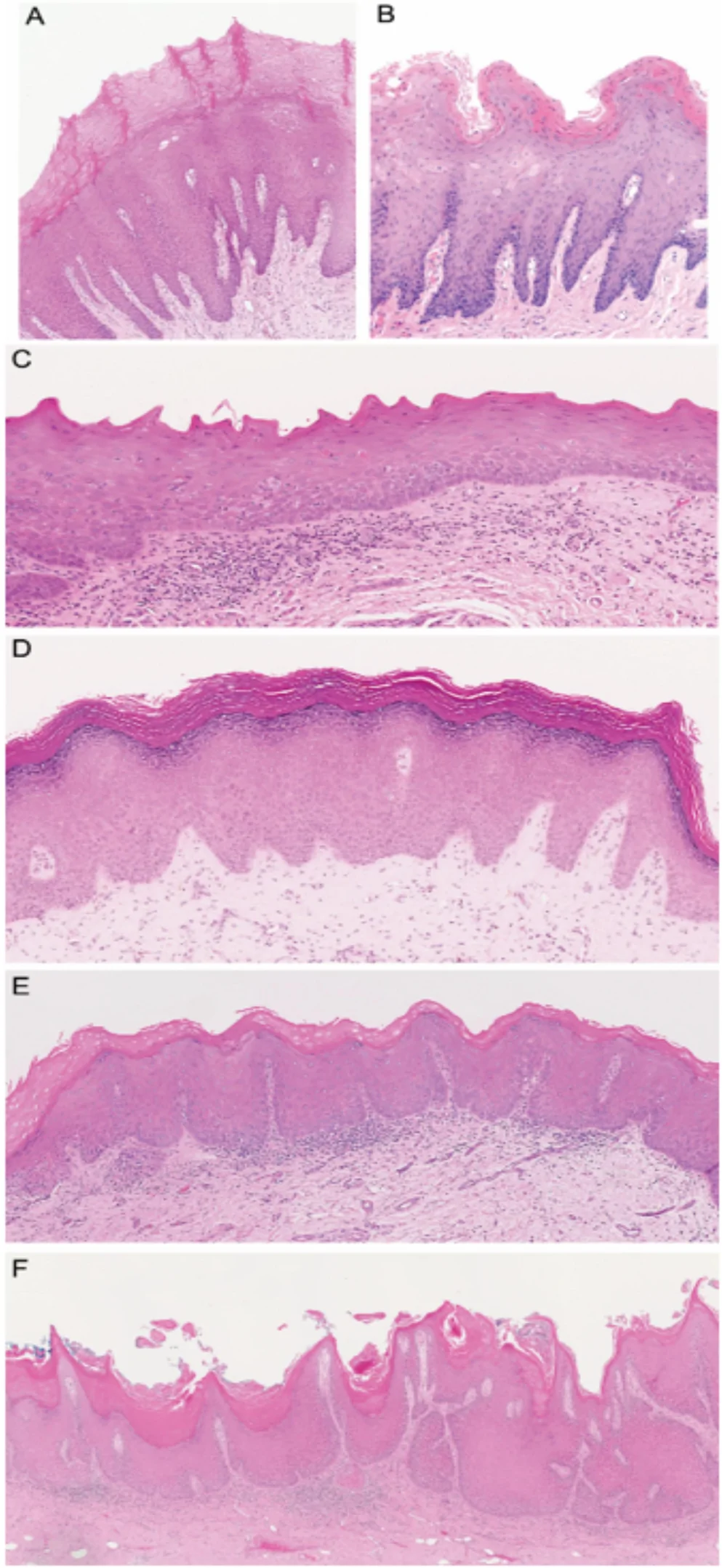
Verrucous pattern. The range of patterns in verrucous pattern dysplasia and some mimics. **A**. Chevron keratinisation, a pattern associated with tobacco smoking but not considered a feature of dysplasia. **B**. Undulating surface keratin lacking other features of verrucous pattern and of no specific significance. **C**. Features suspicious of verrucous pattern. There is some surface undulation and peaking of a thin parakeratin layer and premature keratinisation but full features of the verrucous pattern are lacking and the lesion is of keratinising (differentiated) type. **D**. A fully developed verrucous pattern dysplasia with only architectural features. There is undulating or peaking thick orthokeratin aligned to the rete process pattern with premature keratinisation. Such lesions are usually graded as mild dysplasia with a verrucous pattern. **E**. Fully developed verrucous pattern of peaking also and parakeratin, the peaks are lined with dermal papillae and with broad rounded or blunt ended rete processes showing premature keratinisation throughout. In this example there is also a light immune response. **F**. Verrucous pattern dysplasia starting to develop a mixed verrucous and papillary architecture and with additional features of reduced cohesion in the suprabasal layers and expansion of the proliferating compartment together with irregular sometimes apparently detached islands of epithelium and budding of rete processes. Keratin dipping into rete processes resembling keratin plugging may be prominent. At higher power mitotic figures are fairly frequent and these additional features would increase the grade to severe. Note that though these appearances form a spectrum of developing severity they are not necessarily indicative of progression over time in any one lesion

This pattern is characterised by an exophytic, verruciform architecture (not just keratin peaking) with broad, blunt pushing rete processes aligned with peaking of surface keratin (Fig. 3). Supporting features include church-spire surface keratosis and minimal or no cytological atypia with preserved basal polarity [30–33].

##### Clinical Context

The verrucous pattern is associated with, but not in itself diagnostic of, PVL; recognition of this architectural phenotype must be contextualised within the clinical presentation and correlated with established PVL diagnostic criteria [44]. In the specific clinical context of PVL characterised by multifocal and progressive disease, lesions demonstrate high rates of malignant transformation (44–46% in systematic reviews; [30–32] exceeding 60% in series with longer follow-up [33]). Solitary verrucous lesions without clinical features of PVL carry a substantially lower risk, though the evidence base for precise rate estimates in this subgroup remains limited. The recognition of a verrucous architectural phenotype within a multifocal clinical setting is therefore a significant indicator of high biological risk that substantially exceeds the impression conveyed by cytological grade alone.

##### Grading and Risk Communication

Conventional WHO grading frequently performs poorly for verrucous lesions because cytological atypia is often minimal. This leads to “mild dysplasia” or “hyperkeratosis” diagnoses that substantially understate the actual risk of progression. Critically, the cytological grade must not be reported in isolation for verrucous pattern lesions. The architectural phenotype appears to constitute an important risk indicator and should be explicitly communicated alongside grade in the diagnostic report. Recognition of a verrucous pattern should prompt multidisciplinary discussion regardless of assigned cytological grade [30–32]. The high transformation rates cited apply to lesions arising within the established clinical spectrum of PVL and should not be extrapolated to verrucous morphology in isolation; the architectural phenotype is a risk indicator that requires clinical contextualisation rather than an autonomous high-risk diagnosis. The term “verrucous hyperplasia” should be avoided as this designation implies a non-dysplastic process. True verrucous architecture (not just keratin peaking) associated with any degree of epithelial atypia (or none) constitutes verrucous pattern dysplasia [34, 35].

#### Papillomatous Pattern

##### Defining Features

**Fig. 4:**
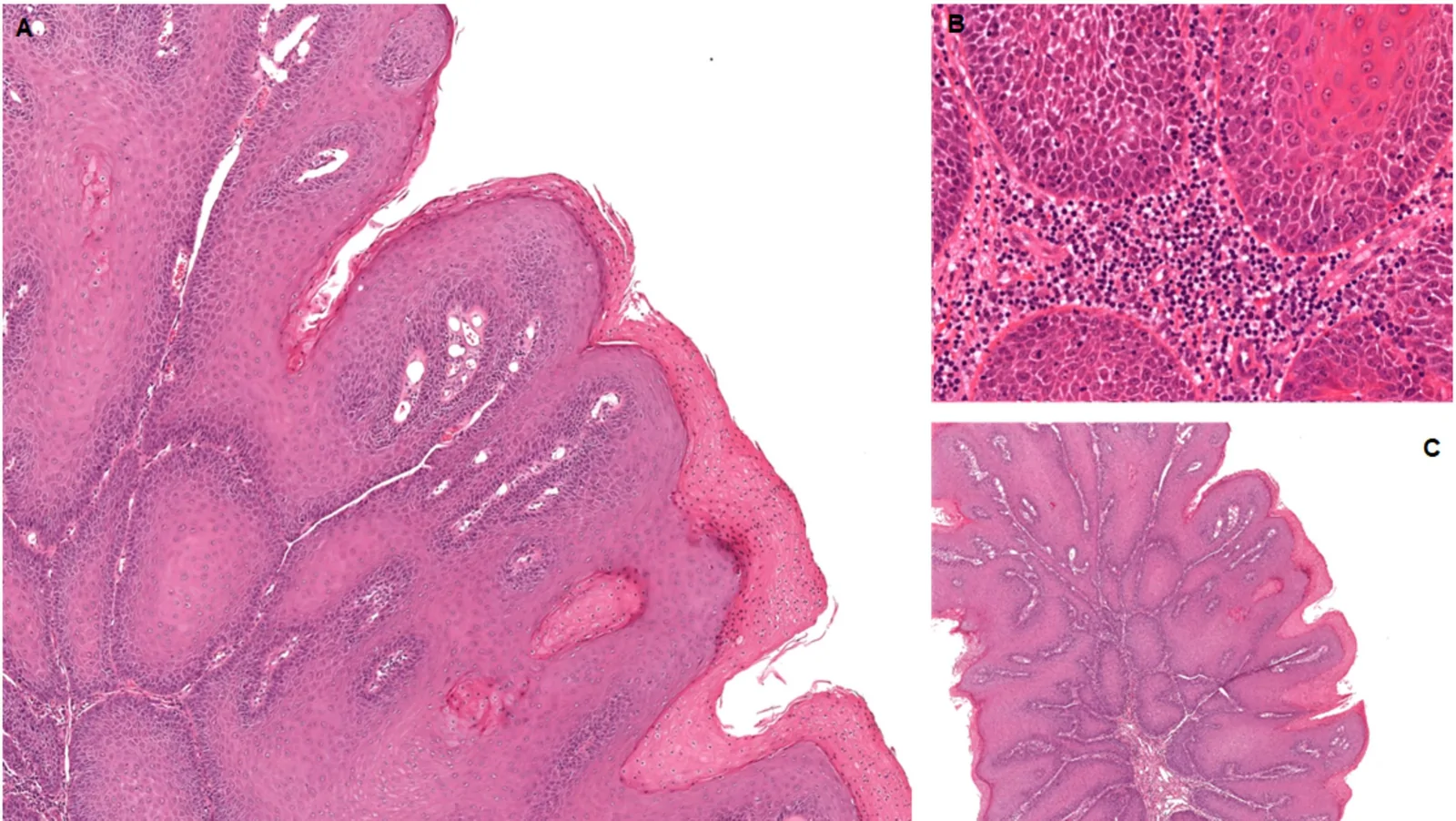
Papillomatous pattern. **A**–**C**: Papillary fronds with thin fibrovascular cores; variable atypia and premature keratinisation. Surface morphology may resemble squamous papilloma; **B**-endophytic growth and basal atypia are key discriminating features. Sampling error on incisional biopsy is a recognised pitfall

The papillomatous pattern is characterised by an exophytic or papillary growth architecture, featuring thin, delicate fibrovascular cores within the epithelial projections (Fig. 4). The degree of cytological atypia is variable but is frequently most pronounced at the basal aspects of the papillae, with relatively preserved maturation towards the surface [32].

##### Clinical Context and Diagnostic Considerations

The papillomatous pattern should be distinguished from squamous papilloma, which demonstrates orderly epithelial maturation, absent or minimal cytological atypia, no dyskeratosis, and lacks endophytic growth. In contrast, papillomatous dysplasia shows basal atypia at the bases of the papillary projections, dyskeratosis, and endophytic growth components, and may demonstrate increased mitotic activity. Where these discriminating features might be absent on an incisional biopsy, sampling error must be considered as the epithelium may be several millimetres thick and specimens may only capture the hyperkeratotic tips of the papillary projections, failing to represent underlying dysplastic changes at the base of the lesion [32]. Where clinically feasible, complete excisional biopsy may be considered to exclude occult invasive carcinoma or higher-grade dysplasia.

#### HPV-Associated Pattern

##### Defining Features

**Fig. 5:**
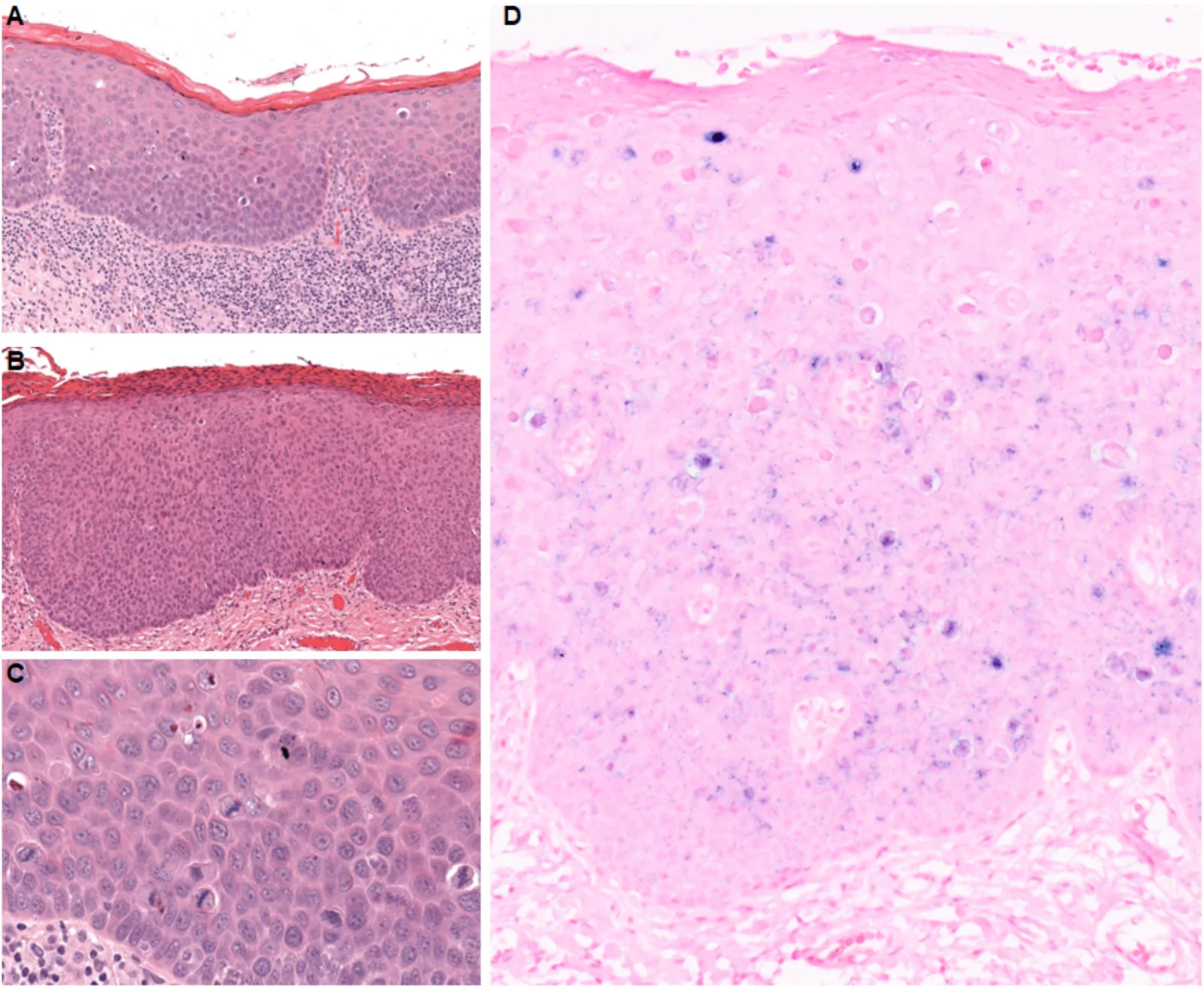
HPV-associated pattern: **A**–**C** H&E: acanthotic epithelium; expanded basaloid proliferative compartment; diffuse atypia at all levels; frequent atypical and suprabasal mitoses; karyorrhectic nuclei. **D** ISH: nuclear positivity for high-risk HPV confirming viral aetiology. Diagnosis requires ancillary confirmation: HPV ISH is mandatory; block-positive p16 immunohistochemistry may serve as a screening indicator but is insufficient alone for diagnosis in the oral cavity

The HPV-associated pattern is characterised by acanthotic epithelium, often thick, with marked expansion of the basaloid/proliferative compartment and reduced surface maturation. Diffuse cytological atypia is present throughout the full epithelial thickness, with frequent atypical mitoses at all levels and karyorrhectic nuclei scattered through the basaloid layers. The cells within the expanded compartment show relatively limited pleomorphism but the atypia is evident from the disordered architecture, frequent abnormal mitoses, and apoptotic debris (Fig. 5).

##### Ancillary Testing

Definitive identification of HPV-associated dysplasia requires confirmation of high-risk HPV by in situ hybridisation (ISH) (Fig. 5). p16 immunohistochemistry showing diffuse, strong nuclear and cytoplasmic expression (block positivity) serves as a useful screening marker to guide ancillary testing but cannot be used as a standalone diagnostic criterion [48] because p16 overexpression in oral dysplasia may reflect non-HPV-driven cell cycle dysregulation. Its specificity for HPV-associated disease is substantially lower than in the oropharynx, and HPV ISH confirmation should therefore be performed before assigning an HPV aetiology [48]. Viral replication occurs in a proportion of these lesions but is usually focal; typical koilocytic change is not a consistent feature of HPV-associated oral dysplasia and should not be expected or required for diagnosis.

##### Anatomical, Prognostic Context, and Grading

True HPV-associated dysplasia is extremely uncommon within the oral cavity proper, representing a small minority of cases [48, 49]. It has been proposed that all HPV-associated dysplasia should be graded as high-grade (severe) on the basis of its morphological similarity to severe basaloid/conventional dysplasia in routine staining. Whilst this reflects the expanded, atypical, poorly differentiating phenotype, formal evidence-based grading criteria specific to this pattern have not been validated, and current evidence does not support modification of management on the basis of HPV association alone for oral cavity lesions.

## Grading Within Pattern

Once the dominant pattern is identified, dysplasia should be graded using existing WHO or binary criteria within that morphological context. This acknowledges that identical grades may carry different implications depending on pattern.

The practical implications of grading within pattern vary by phenotype. Low-grade keratinising dysplasia warrants closer surveillance than cytologically equivalent conventional (basaloid) dysplasia, given its documented association with under-recognised high-risk disease, and should trigger MDT discussion [10, 23, 26]. Verrucous-pattern lesions require the risk-communication approach set out in Sect. "Verrucous pattern", since “low-grade” or “mild” designations otherwise risk falsely reassuring treating clinicians and surgeons [30–33]. High-grade conventional (basaloid)-pattern dysplasia with bulbous rete and cohesion loss constitutes the highest-risk category within that phenotype and warrants consideration of excision where clinically feasible [22].

When two distinct patterns are identified at the same epithelial level within a single biopsy, both should be reported and management guided by the higher-risk pattern. The coexistence of dysplasia with an underlying condition such as oral lichen planus or actinic cheilitis should be explicitly stated in the diagnostic report. In these contexts, pattern recognition remains applicable but the interpretive threshold for architectural features may need adjustment where inflammation is extensive, and close clinicopathological correlation is essential.

This approach adds critical biological context to the existing WHO criteria and can inform therapeutic decision-making and surveillance intensity.

## Convergent Evidence From Computational Pathology

Independent computational pathology studies provide convergent support for a pattern-based view of OED. Feature-specific prognostic models and machine-learning analyses consistently prioritise architectural disturbance, epithelial organisation, and spatial relationships as dominant predictors of malignant transformation, rather than grade alone [21, 22, 38].

In recent studies, AI analysis using whole-slide imaging identified peri-epithelial lymphocyte density (concordance index 0.73, *p* < 0.05) and intra-epithelial lymphocyte scores (concordance index 0.67, *p* < 0.001) as novel prognostic markers not explicitly incorporated into WHO criteria [38, 39]. These immune microenvironment features frequently accompany verrucous and keratinising/differentiated pattern dysplasia, reinforcing the biological distinctiveness of these phenotypes [26, 38]. Transcriptional analyses identifying distinct immune profiles in high-risk OED provide additional, convergent evidence for the biological relevance of the stromal immune signal [50, 51].

The histological features prioritised by AI models including bulbous rete processes, cohesion loss, and stromal inflammation patterns, align closely with morphologically recognisable patterns such as conventional/basaloid proliferation, keratinising differentiation, and lichenoid stromal responses [21, 22, 38]. Emerging longitudinal genetic data further support this view, with recent evidence demonstrating that distinct clonal trajectories are maintained as oral lesions progress towards carcinoma, supporting the hypothesis that distinct morphological patterns may reflect relatively stable biological trajectories in oral carcinogenesis [52].

More recently, fully automated and explainable AI algorithms have been developed that predict malignant transformation directly from H&E-stained whole slide images without requiring manual feature scoring. Shephard et al. developed a pipeline leveraging automated segmentation of nuclei and epithelium alongside interpretable morphological and spatial features to assign an oral malignant transformation risk score, demonstrating strong prognostic performance across a multicentre cohort [53]. An independently developed AI pipeline subsequently validated the approach in a separate cohort, corroborating the centrality of epithelial architecture and immune cell distribution as predictive signals [54]. Broader evidence that AI can stratify recurrence and malignant progression risk in OPMDs following excision provides additional translational context for these developments [55]. These algorithmic advances represent important progress towards clinically deployable tools, though prospective validation in routine diagnostic settings remains essential.

It should be acknowledged, however, that these computational findings remain in the research domain. The models have not yet achieved independent, prospective validation at the level required for routine clinical deployment, and whilst the association between lichenoid inflammation and dysplastic progression is statistically robust, a causal relationship has not been formally established. The convergence with pattern-based assessment is therefore presented as hypothesis-supporting evidence requiring further validation, not as confirmation of the framework’s clinical utility.

## Future Work

This proposal should be regarded as hypothesis-driven and requires formal validation before wider adoption. Key priorities are: (1) formal definition of grading criteria within each pattern, specifying the histological thresholds and feature combinations that determine mild, moderate and severe dysplasia within the conventional/basaloid, keratinising, verrucous, papillomatous and HPV-associated contexts as without agreed criteria, no reproducible validation study can be designed or conducted; (2) assessment of inter- and intra-observer reproducibility of pattern recognition across multiple centres and experience levels, including repeat evaluation after washout to determine whether pattern assignment is more stable over time than conventional grading; [13, 15] (3) evaluation of prognostic performance of pattern-anchored grading against WHO and binary grading in independent cohorts with long-term follow-up, with minimum 5 years for conventional dysplasia and 10–15 years for PVL-spectrum lesions; (4) determination of whether pattern descriptors reduce under-diagnosis and improve risk communication in routine practice, particularly in chronic inflammatory contexts where conventional grading is most vulnerable; and (5) investigation of the molecular underpinnings of each pattern to provide biological validation of the proposed archetypes.

Supporting deliverables required for validation include an image-anchored exemplar set illustrating each pattern across WHO grades, a structured reporting template compatible with existing WHO terminology, and a prospective registry capturing pattern, grade, treatment, and outcomes data. Coordination within COST Action INTERCEPTOR Working Group 2 provides an appropriate collaborative platform, though no clinical implementation is advocated until validation is complete.

## Limitations

Several important limitations must be acknowledged. The proposed patterns are consensus-derived and require validation [10, 11, 21–23, 26, 27, 29, 32]. Inter-observer reproducibility for pattern assignment, whilst hypothesised to exceed conventional grading due to architectural anchors, has not been formally demonstrated in multicentre studies. This applies with particular force to the dominance-assignment step described in Sect. "Architecture-predominant dysplasia and the under-recognition problem": because most lesions show some degree of mixed morphology, the criteria by which one pattern is judged dominant over another are themselves untested, and it remains possible that this step reintroduces, rather than removes, a source of inter-observer disagreement. Pattern recognition may be more intuitive than assessing individual features, but this assumption requires empirical testing [13, 15].

The inherent tension between cytological grade and biological risk is particularly acute for verrucous pattern lesions. A report of “verrucous pattern dysplasia, low-grade” risks being interpreted by treating clinicians and surgeons as indicating low overall risk, which would be incorrect. The framework depends on clinicians understanding that grade reflects cytological severity *within* the assigned pattern, and that pattern and grade together constitute the complete risk assessment. Until this distinction is widely appreciated, there is a risk of grade-based clinical decisions that fail to reflect the true biological risk. Educational materials accompanying any implementation of this framework should address this explicitly.

The temporal stability of these patterns during lesion evolution is not yet fully elucidated. Morphological features may transition over time, and serial biopsies might reveal phenotypic shifts that add complexity to longitudinal risk assessment.

Cohort sizes required to fully validate this framework are considerable. Evaluating five distinct patterns across three grades yields fifteen potential subgroups; achieving sufficient statistical power to determine transformation risk for each likely necessitates high-volume longitudinal data. This is particularly relevant for infrequent phenotypes such as the HPV-associated and papillomatous patterns. These requirements underscore the value of multicentre collaborative initiatives in aggregating the large-scale datasets needed to refine these risk-stratification models.

Biopsy sampling error is inevitable. Small incisional biopsies may sample non-representative areas or miss patterns present elsewhere in the lesion (particularly verrucous and papillomatous), introducing misclassification bias that attenuates apparent pattern-outcome associations [32].

Despite these limitations, the convergence of evidence from feature-based prognostic models, [21, 22, 24, 25] under-recognition studies, [9, 10, 23, 26] PVL outcomes, [30–33] and computational pathology [38, 39] provides a compelling rationale for formal validation of pattern-anchored grading.

## Conclusions

The central challenge in OED assessment lies not only in grading severity but in reliably recognising dysplasia across its full morphological spectrum. Current WHO and binary grading systems remain essential; however, they do not fully address real-world diagnostic variability, particularly for architecture-predominant lesions with limited cytological atypia, and they do not provide explicit guidance on communicating pattern-related risk where cytological grade alone understates biological risk [9, 10, 13, 26].

Explicit morphological pattern recognition, used alongside conventional grading, offers a pragmatic and biologically grounded refinement. By anchoring dysplasia recognition before grading, narrowing the range of features to be evaluated within each pattern, and communicating pattern as an independent risk signal, there is potential to reduce false-negative diagnoses, improve interpretive consistency, and enhance communication of risk to clinicians managing OPMDs and OED.

The proposed framework operates within existing WHO classifications and clinical pathways. It does not require abandonment of established terminology or adoption of novel grading schemes. Instead, it makes explicit what expert pathologists already do implicitly: recognise patterns, contextualise findings, and grade within morphological context.

Robust validation is required to determine the prognostic utility, reproducibility, and clinical value of pattern-anchored grading. This represents an appropriate next step for the oral pathology and oral medicine communities.

## Data Availability

No datasets were generated or analysed during the current study.

## References

[1] Warnakulasuriya S, Kujan O, Aguirre-Urizar JM, Bagan JV, Gonzalez-Moles MA, Kerr AR et al (2021) Oral potentially malignant disorders: a consensus report from an international seminar on nomenclature and classification. Oral Dis 27:1862–1880 33128420 10.1111/odi.13704

[2] Warnakulasuriya S, Reibel J, Bouquot J, Dabelsteen E (2008) Oral epithelial dysplasia classification systems: predictive value, utility, weaknesses and scope for improvement. J Oral Pathol Med 37:127–133 18251935 10.1111/j.1600-0714.2007.00584.x

[3] Gopinath D, On C, Veettil SK, Tilakaratne WM (2025) Is binary grading better than WHO system for grading epithelial dysplasia? A systematic review and meta-analysis. Oral Dis 31:59–68 39503340 10.1111/odi.15160

[4] Ranganathan K, Kavitha L, Sharada P, Bavle RM, Rao RS, Pattanshetty SM et al (2020) Intra-observer and inter-observer variability in two grading systems for oral epithelial dysplasia: a multi-centre study in India. J Oral Pathol Med 49:948–955 32516857 10.1111/jop.13056

[5] Hankinson P, Mahmood H, Walsh H, Speight PM, Khurram SA (2024) Demystifying oral epithelial dysplasia: a histological guide. Pathology 56:11–23 38030478 10.1016/j.pathol.2023.10.002

[6] Nevanpää M, Terava AE, Laine HK, Rautava J (2022) Malignant transformation of oral epithelial dysplasia in Southwest Finland. Sci Rep 12:8315 35585112 10.1038/s41598-022-12441-9 PMC9117212

[7] Aguirre-Urízar JM, Lafuente-Ibáñez de Mendoza I, Warnakulasuriya S (2021) Malignant transformation of oral leukoplakia: systematic review and meta-analysis of the last 5 years. Oral Dis 27:1881–1895 33606345 10.1111/odi.13810

[8] Iocca O, Sollecito TP, Alawi F, Weinstein GS, Newman JG, Dr Virgilio A et al (2020) Potentially malignant disorders of the oral cavity and oral dysplasia: a systematic review and meta-analysis of malignant transformation rate by subtype. Head Neck 42:539–555 31803979 10.1002/hed.26006

[9] Odell EW, Kujan O, Warnakulasuriya S, Sloan P (2021) Oral epithelial dysplasia: recognition, grading and clinical significance. Oral Dis 27:1947–1976 34418233 10.1111/odi.13993

[10] Wils LJ, Poell JB, Evren I, Koopman MS, Bronus EREA, Visscher JGAM et al (2020) Incorporation of differentiated dysplasia improves prediction of oral leukoplakia at increased risk of malignant progression. Mod Pathol 33:1033–1040 31896811 10.1038/s41379-019-0444-0 PMC7280084

[11] Kallarakkal TG, Zaini ZM, Ghani WMN, Karen-Ng LP, Siriwardena BSMS, Cheong SC et al (2024) Calibration improves the agreement in grading oral epithelial dysplasia: findings from a National workshop in Malaysia. J Oral Pathol Med 53:53–60 38081145 10.1111/jop.13501

[12] Müller S, Tilakaratne WM (2022) Update from the 5th edition of the World Health Organisation classification of head and neck tumours: Tumours of the oral cavity and mobile tongue. Head Neck Pathol 16:54–62 35312982 10.1007/s12105-021-01402-9 PMC9018914

[13] Marques L-C, P-dP M, Goncalves L-S, Cunha K-S, Junior A-S, Conde D-C (2024) Challenges in the assessment of epithelial dysplasia in oral lichen planus and oral lichenoid lesion: Inter and intra-observer variability of the WHO criteria and binary system. J Clin Exp Dent 16(1):e62–e70 38314343 10.4317/jced.61073 PMC10837801

[14] El-Naggar AK, Chan JKC, Grandis JR, Takata T, Slootweg PJ (eds) (2017) WHO classification of head and neck tumours, 4th edition. IARC Press, Lyon

[15] Kujan O, Khattab A, Oliver RJ, Roberts SA, Thakker N, Sloan P (2007) Why oral histopathology suffers inter-observer variability on grading oral epithelial dysplasia: an attempt to understand the sources of variation. Oral Oncol 43:224–231 16931119 10.1016/j.oraloncology.2006.03.009

[16] Nankivell P, Williams H, Matthews P, Suortamo S, Snead D, McConkey C et al (2013) The binary oral dysplasia grading system: validity testing and suggested improvement. Oral Surg Oral Med Oral Pathol Oral Radiol 115:87–94 23217539 10.1016/j.oooo.2012.10.015

[17] Landis JR, Koch GG (1977) The measurement of observer agreement for categorical data. Biometrics 33:159–174 843571

[18] Smith CJ, Pindborg JJ (1969) Histological grading of oral epithelial atypia by the use of photographic standards. WHO international reference centre for oral precancerous lesions

[19] Speight PM, Khurram SA, Kujan O (2018) Oral potentially malignant disorders: risk of progression to malignancy. Oral Surg Oral Med Oral Pathol Oral Radiol 125:612–627 29396319 10.1016/j.oooo.2017.12.011

[20] Chaturvedi AK, Udaltsova N, Engels EA, Katzel JA, Yanik EL, Katki HA et al (2020) Oral leukoplakia and risk of progression to oral cancer: a population-based cohort study. J Natl Cancer Inst 112:1047–1054 31860085 10.1093/jnci/djz238 PMC7566351

[21] Mahmood H, Bradburn M, Rajpoot N, Islam NM, Kujan O, Khurram SA (2022) Prediction of malignant transformation and recurrence of oral epithelial dysplasia using architectural and cytological feature specific prognostic models. Mod Pathol 35:1151–1159 35361889 10.1038/s41379-022-01067-x PMC9424112

[22] Mahmood H, Bradburn M, Islam NM, Kujan O, Rajpoot N, Santos-Silva AR et al (2026) Independent multicentre validation of the ‘six-point’ model for malignant transformation risk in oral epithelial dysplasia. Oral Dis 32(5):1273–1282. 10.1111/odi.70173 41450095 PMC13365013

[23] Hankinson P, Clark M, Walsh H, Khurram SA (2025) A head-to-head comparison of four grading systems for oral epithelial dysplasia. Histopathology 86:933–941 39688116 10.1111/his.15400 PMC11964578

[24] Soares AB, Warnakulasuriya S, da Silveira Terra Junqueira L, de Freitas ALS, Schneider A, Silva ARS et al (2025) Risk assessment of oral leukoplakia by individual dysplasia features. J Oral Pathol Med 54:537–546 40432218 10.1111/jop.13633

[25] Rodrigues AZ, Laureano NK, Maraschin BJ, da Silva AD, da Silva VP, Rados PV et al (2025) Diagnostic criteria for oral epithelial dysplasia: predicting malignant transformation. Head Neck Pathol 19:21 39918668 10.1007/s12105-025-01754-6 PMC11806176

[26] Wils LJ, Poell JB, Peferoen LAN, Evren I, Brouns ER, de Visscher JGAM et al (2023) The role of differentiated dysplasia in the prediction of malignant transformation of oral leukoplakia. J Oral Pathol Med 52(10):930–938 37749621 10.1111/jop.13483

[27] Kim E, Chung M, Jeong HS, Baek CH, Cho J (2022) Histological features of differentiated dysplasia in the oral mucosa: a review of oral invasive squamous cell carcinoma cases diagnosed with benign or low-grade dysplasia on previous biopsies. Hum Pathol 126:45–54 35597368 10.1016/j.humpath.2022.05.010

[28] Heller DS, Day T, Allbritton JI, Scurry J, Radici G, Welch K et al (2021) Diagnostic criteria for differentiated vulvar intraepithelial neoplasia and vulvar aberrant maturation. J Low Genit Tract Dis 25(1):57–70 33105449 10.1097/LGT.0000000000000572 PMC7748053

[29] Pinto AP, Miron A, Yassin Y, Monte N, Woo TYC, Mehra KK et al (2010) Differentiated vulvar intraepithelial neoplasia contains Tp53 mutations and is genetically linked to vulvar squamous cell carcinoma. Mod Pathol 23:404–412 20062014 10.1038/modpathol.2009.179

[30] Mohideen K, Ghosh S, Krithika C, Ali-Hassan M, Chole R, Dhungel S (2025) Malignant transformation of proliferative verrucous leukoplakia-systematic review & meta-analysis. BMC Oral Health 25(1):175 39893387 10.1186/s12903-025-05565-7 PMC11786437

[31] Alabudalaly L, Villa A, Chen T, Kerr A, Ross N, Abreu Alves F et al (2022) Characterization of initial/early histologic features of proliferative leukoplakia and correlation with malignant transformation: a multicenter study. Mod Pathol 35:1034–1044 35184151 10.1038/s41379-022-01021-x

[32] Palaia G, Bellisario A, Pampena R, Pippi R, Romeo U (2021) Oral proliferative verrucous leukoplakia: progression to malignancy and clinical implications. Systematic review and meta-analysis. Cancers (Basel) 13(16):4085 34439238 10.3390/cancers13164085 PMC8391406

[33] Müller S (2018) Oral epithelial dysplasia, atypical verrucous lesions and oral potentially malignant disorders: focus on histopathology. Oral Surg Oral Med Oral Pathol Oral Radiol 125:591–602 29606637 10.1016/j.oooo.2018.02.012

[34] Chiu SF, Ho CH, Chen YC, Wu LW, Chen YL, Wu JH et al (2021) Malignant transformation of oral potentially malignant disorders in Taiwan: an observational nationwide population database study. Med (Baltimore) 100(9):e24934 10.1097/MD.0000000000024934 PMC7939230 33655959

[35] Maulana Y, Wu M-H, Chiang W-F, Hsiao W-P, Mong L-C, Wang W-C (2026) A pilot genome-wide association study of malignant transformation of oral verrucous hyperplasia. Oral Dis 32(1):80–88 40698521 10.1111/odi.70037

[36] Goodson ML, Sloan P, Robinson CM, Cocks K, Thomson PJ (2015) Oral precursor lesions and malignant transformation-who, where, what, and when? Br J Oral Maxillofac Surg 53:831–835 26388071 10.1016/j.bjoms.2015.08.268

[37] Stojanov IJ, Omari J, Akeel I, Sultan AS, Woo SB (2024) Oral epithelial dysplasia with lymphocytic immune response: clinicopathological characterisation of 44 cases. Histopathology 85:40–50 38497348 10.1111/his.15171

[38] Bashir RMS, Shephard AJ, Mahmood H, Azarmehr N, Raza SEA, Khurram SA et al (2023) A digital score of peri-epithelial lymphocytic activity predicts malignant transformation in oral epithelial dysplasia. J Pathol 260:431–442 37294162 10.1002/path.6094 PMC10952946

[39] Shephard AJ, Mahmood H, Raza SEA, Khurram SA, Rajpoot NMl (2025) A novel AI-based score for assessing the prognostic value of intra-epithelial lymphocytes in oral epithelial dysplasia. Br J Cancer 132:168–179 39616233 10.1038/s41416-024-02916-z PMC11747091

[40] Chaudhary M, Gadbail AR, Vidhale G, Gadbail MPM, Gondivkar SM, Gawande M et al (2012) Comparison of myofibroblasts expression in oral squamous cell carcinoma, verrucous carcinoma, high risk epithelial dysplasia, low risk epithelial dysplasia and normal oral mucosa. Head Neck Pathol 6:305–313 22392407 10.1007/s12105-012-0335-x PMC3422591

[41] Varghese SS, Sarojini SB, George GB, Vinod S, Mathew P, Babu A et al (2015) Evaluation and comparison of the biopathology of collagen and inflammation in the extracellular matrix of oral epithelial dysplasias and inflammatory fibrous hyperplasia using Picrosirius Red Stain and polarising microscopy: a preliminary study. J Cancer Prev 20:275–280 26734590 10.15430/JCP.2015.20.4.275 PMC4699755

[42] Coletta RD, Salo T (2018) Myofibroblasts in oral potentially malignant disorders: is it related to malignant transformation? Oral Dis 24:84–88 29480603 10.1111/odi.12694

[43] Bouquot JE, Speight PM, Farthing PM (2006) Epithelial dysplasia of the oral mucosa-Diagnostic problems and prognostic features. Diagn Histopathol 12:11–21

[44] Thompson LDR, Fitzpatrick SG, Müller S, Eisenberg E, Upadhyaya JD, Lingen MW et al (2021) Proliferative verrucous leukoplakia: an expert consensus guideline for standardized assessment and reporting. Head Neck Pathol 15:572–587 33415517 10.1007/s12105-020-01262-9 PMC8134585

[45] Rohilla M, Pandiar D, Krishnan RP (2025) Correlation of newly added dysplastic features in the latest WHO diagnostic criteria of oral epithelial dysplasia with malignant transformation and recurrence. Head Neck Pathol 19:98 40773046 10.1007/s12105-025-01835-6 PMC12332147

[46] Arsenic R, Kurrer MO (2013) Differentiated dysplasia is a frequent precursor or associated lesion in invasive squamous cell carcinoma of the oral cavity and pharynx. Virchows Arch 462:609–617 23588556 10.1007/s00428-013-1412-6

[47] Becker AS, Holm M, Liese J, Engel N, Zimpfer AH (2024) Diagnosis of differentiated dysplasia as a variant of oral epithelial dysplasia. Oral Dis 30:4185–4194 38191851 10.1111/odi.14846

[48] Woo SB (2019) Oral epithelial dysplasia and premalignancy. Head Neck Pathol 13:423–439 30887394 10.1007/s12105-019-01020-6 PMC6684678

[49] Speight PM (2007) Update on oral epithelial dysplasia and progression to cancer. Head Neck Pathol 1:61–68 20614284 10.1007/s12105-007-0014-5 PMC2807503

[50] Gan CP, Lee BKB, Lau SH, Kallarakkal TG, Zaini ZM, Lye BKW et al (2022) Transcriptional analysis highlights three distinct immune profiles of high-risk oral epithelial dysplasia. Front Immunol 13:954567 36119104 10.3389/fimmu.2022.954567 PMC9479061

[51] Flores-Hidalgo A, Phero J, Steward-Tharp S, Williamson M, Paquette D, Krishnan D et al (2024) Immunophenotypic and gene expression analyses of the inflammatory microenvironment in high-grade oral epithelial dysplasia and oral lichen planus. Head Neck Pathol 18:17 38456941 10.1007/s12105-024-01624-7 PMC10923754

[52] Wils LJ, Poell JB, Brink A, Peferoen LA, Evren I, Bronus ER et al (2025) New insights into the genetic progression of cancer through longitudinal analysis of oral lesions. J Pathol 267:155–167 40728388 10.1002/path.6449 PMC12438000

[53] Shephard AJ, Bashir RMS, Mahmood H, Jahanifar M, Minhas F, Raza SEA, et al (2024) A fully automated and explainable algorithm for predicting malignant transformation in oral epithelial dysplasia. npj Precis Oncol 8:137 10.1038/s41698-024-00624-8 PMC11213925 38942998

[54] Shephard AJ, Mahmood H, Raza SEA, Arauo ALD, Santos-Silva AR, Lopes MA et al (2025) Development and validation of an artificial intelligence-based pipeline for predicting oral epithelial dysplasia malignant transformation. Commun Med (Lond) 5:186 40394272 10.1038/s43856-025-00873-z PMC12092575

[55] Adeoye J, Su YX (2026) Artificial intelligence for predicting post-excision recurrence and malignant progression in oral potentially malignant disorders: a retrospective cohort study. Int J Surg 112(1):1392–1401 41056008 10.1097/JS9.0000000000003592 PMC12825756

